# Supplementary material for: Ionic Nanocomplexes of Hyaluronic Acid and Polyarginine to Form Solid Materials: A Green Methodology to Obtain Sponges with Biomedical Potential
Source: Nanomaterials (Basel). 2019 Jun 29;9(7):944. doi: 10.3390/nano9070944 (PMC6669755; doi:10.3390/nano9070944)
Supplement: Supplementary file 1 [file nanomaterials-09-00944-s001.zip › nanomaterials-514027 - supplemetary materials update/nanomaterials-514027-supplementary materials.pdf]

Supplementary information section:

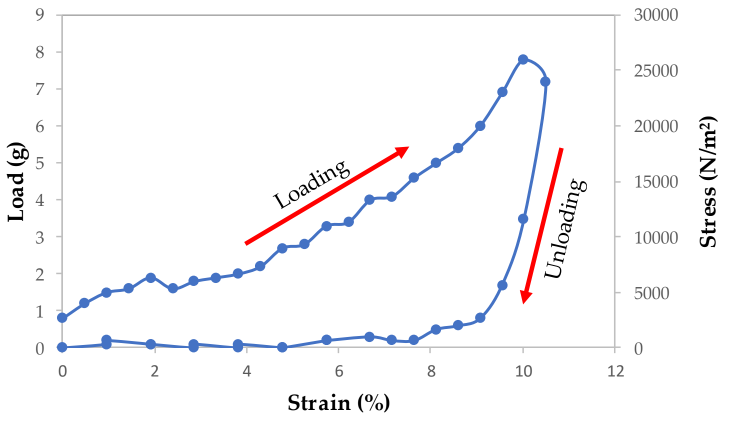

Figure S1. Typical strain-stress behavior of the sponges analyzed.

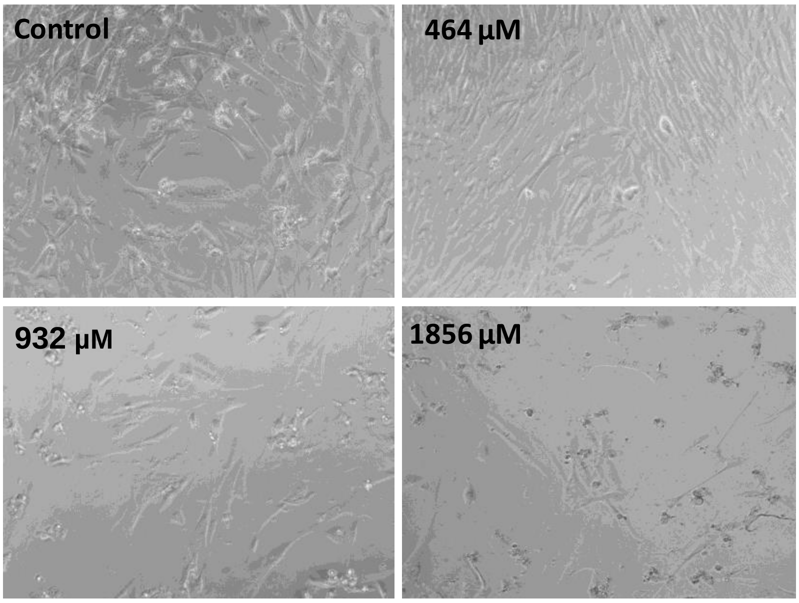

Figure S2. Phase contrast microphotographs of fibroblast (RMF-EG cell line) exposed to different doses of sponges of LMWHA/PArg = 2.4.

Methodology: RMF-EG cells (5.000) were seeded in 96 well plates. After letting the cells adhere to the plates (2 hrs), they were incubated with different sponges concentrations (1856 µM; 932µM; 464 µM and without sponges) for 72 hrs. Pictures obtained with phase contrast microscope are shown at 20× magnification.
